# Risk of second primary malignancy in adults with pulmonary high-grade neuroendocrine carcinoma (HGNEC)

**DOI:** 10.1186/s12885-020-07224-2

**Published:** 2020-08-03

**Authors:** Xiaomin Wu, Xiaojing Zhang, Leilei Tao, Ping Chen

**Affiliations:** 1 Department of Oncology, Yancheng No.1 People’s Hospital, the Affiliated Hospital of Nanjing University, 166 Yulong West Road, Yancheng, 224200 People’s Republic of China; 2 Department of Gynecologic Oncology, Zhejiang Cancer Hospital, Hangzhou, 310000 People’s Republic of China

## Abstract

**Background:**

Pulmonary high-grade neuroendocrine carcinoma (HGNEC) has a rising incidence of developing second primary malignancies (SPMs). This study is the first population-based analysis to quantify the SPM risks among survivors of lung HGNEC.

**Methods:**

We used the Surveillance, Epidemiology, and End Results (SEER) database to calculate standardized incidence ratio (SIR) and absolute excess risk (AER) between 2000 and 2016 for patients with pulmonary HGNEC.

**Results:**

The data of 1161 patients with SPMs were retrieved from the SEER database. The ratio of observed/expected number of SPMs in pulmonary HGNEC was 1.53. Solid tumours comprised 91% of all second malignancies in lung HGNEC patients, with the most common cancers reported in the oral cavity and pharynx, the urinary and respiratory systems

**Conclusions:**

Our study observed an increased risk of SPMs among patients with pulmongnancies.

## Background

Lung cancer is one of the most frequently diagnosed cancers and the leading cause of cancer death in the United States [1, 2]. Pulmonary high-grade neuroendocrine carcinoma (HGNEC), including small cell lung cancer (SCLC) and large cell neuroendocrine carcinoma (LCNEC), is a heterogeneous group of poorly differentiated neoplasms and covers 20% of all lung cancers. Remarkably, these two subtypes have relatively similar histological, genetic, and clinical characteristics, such as higher incidence in males and heavy smokers, as well as high mitotic rate and necrosis at histologic examination. It is also widely believed that they have similarly poor overall survival [3].

Cancer survivors have been increasing due to the improvement in diagnostic modalities and treatment of cancers. Second primary malignancy (SPM) is one of the most severe long-term complications in the population of cancer survivors. Several studies have demonstrated that patients with initial primary lung cancer have a higher risk of developing second primary lung cancer [4]. According to research done by Wu and coworkers, the incidence of SPMs among patients with non-small cell lung cancer is about 6.4%. Furthermore, their findings indicated that 50.7% of SPMs occurred during the first year after the diagnosis of non-small cell lung cancer [5]. However, the risk of SPMs following a diagnosis of lung HGNEC remains unclear.

In this context, we aimed to assess the risk of developing SPM in patients with pulmonary HGNEC in the United States utilizing the Surveillance, Epidemiology, and End Results (SEER) database. We obtained the standardized incidence ratio (SIR) of SPM after diagnosis of pulmonary HGNEC between January 2000 and December 2016. The incidence of SPMs stratified by age, sex, race, and latency was also analyzed. Additionally, multivariate Cox regression model was applied to investigate the factors affecting overall survival (OS) and cancer-specific survival (CSS) in patients with SPMs.

## Methods

We obtained data on lung HGNEC patients from the SEER database, which collects cancer incidence and survival data from 18 regional cancer registries. These registries represent about 26% of the U.S. population. Using a 6-month minimum interval, as is required to exclude synchronous primary cancers, we identified cases of histologically confirmed HGNEC with primary site codes (C34.0-main bronchus; C34.1-upper lobe, lung; C34.2-middle lobe, lung; C34.3-lower lobe, lung; C34.8-overlapping lesion of the lung; and C34.9-lung, NOS) and ICD-0-3 Hist/Behav (8002/3: malignant tumour, small cell type; 8013/3: Large-cell neuroendocrine carcinoma; 8041/3: small cell carcinoma, NOS; 8042/3: oat cell carcinoma; 8043/3: small cell carcinoma, fusiform cell; 8044/3: small cell carcinoma, intermediate cell; and 8045/3: combined small cell carcinoma). Patients with no histologically confirmed cancer, and those diagnosed only based on autopsy/death certificate were excluded as were those under the age of 20 years. After the exclusion of patients who did not have active follow-up, 75,877 patients were ultimately eligible for inclusion into the present investigation.

We collected data on patient demographics (age, gender, and ethnicity), treatment (radiotherapy and chemotherapy), HGNECs (cancer site and histological subtype) and survival (survival period, vital status, and cause-specific death classification). We utilized SEER*Stat multiple primary-standardized incidence ratio (MP-SIR) software version 8.3.5 (www.seer.cancer.gov/seerstat), to calculate the SIR and absolute excess risk (AER) for SPM occurrence. An SPM is defined as a metachronous cancer that develops at least 6 months after the first cancer diagnosis, according to the methods used previously. We estimated SIR as the ratio of the number of incident cases of cancers in patients with pulmonary HGNEC to the number of expected cases in the U.S. general population. SIR over 1.0 indicated that more cases were observed than would be expected. AER was calculated as the excess number of SPMs in patients with pulmonary HGNEC per 10,000 person-years at risk. We performed subgroup analyses by further stratifying patients according to their age at diagnosis, gender, ethnicity, calendar-years, and months of follow-up since the diagnosis of cancer.

Multivariate Cox regression analyses, which could identify the associations between different clinical characteristics and survival, were performed to estimate hazard ratios (HRs) and the associated 95% confidence intervals (CIs). All statistical tests were two-sided, and *P* values less than 0.05 were assumed to be significant. Our data were obtained from the SEER program and imported into SPSS software, and data analyses were performed using IBM SPSS statistics for Windows, version 23 (IBM Corp, Armonk, New York, USA).

## Results

Between 2000 and 2016, 75,877 patients were diagnosed with pulmonary HGNEC and met inclusion criteria. 72,381 patients with small cell carcinoma and 3496 patients with large-cell neuroendocrine carcinoma were included. A total of 1161 cases with primary pulmonary HGNEC, including 1022 SCLC and 139 LCNEC, developed a cohort of 1361 SPMs. Among those with SMPs, 979 patients had only 1 SPM, and 182 had more than 2 SPMs. The demographic characteristics of both groups are displayed in Table 1.

**Table 1:** Demographics of patients

	Demography			
	SCLC		LCNEC	
	Number/Median	%/Range	Number/Median	%/Range
**Total patients**	72,381		3496	
Sex				
Male	36,406	50.30%	1928	55.15%
Female	35,975	49.70%	1568	44.85%
Race				
White	63,192	87.30%	2919	83.50%
Black	6284	8.68%	423	12.10%
Other/unknown	2905	4.01%	154	4.41%
**Total patients with SPM**	1022	1.41%	139	3.98%
Sex				
Male	458	44.81%	68	48.92%
Female	564	55.19%	71	51.08%
Race				
White	899	87.96%	113	81.29%
Black	85	8.32%	19	13.67%
Other/unknown	38	3.72%	7	5.04%
**Patients with 1 SPM**	862	1.20%	117	3.35%
Sex				
Male	384	44.55%	57	48.72%
Female	478	55.45%	60	51.28%
Race				
White	755	87.59%	96	82.05%
Black	74	8.58%	15	12.82%
Other/unknown	33	3.83%	6	5.13%
**Patients with 2 or more SPM**	160	0.23%	22	0.63%
Sex				
Male	74	46.25%	11	50%
Female	86	53.75%	11	50%
Race				
White	143	89.38%	17	77.27%
Black	12	7.50%	4	18.18%
Other/unknown	5	3.12%	1	4.55%
**Age for SPM (median)**	64 years	(36-88 years)	66 years	(37-82 years)
**Latency (median)**	3.58 years	(0.5–16.42 years)	3.33 years	(0.67-15 years)
**Follow-up (median)**	5.42 years	(0.5–16.92 years)	5.92 years	(0.67–15.75 years)

### Overall risk of SPM

This cohort had a trend of higher SPM incidence than expected in the general population (SIR 1.53; 95% CI, 1.45 to 1.62; AER 83.11). Site-specific analyses of SIRs indicated the highest risk of malignancy in the acute monocytic leukemia (SIR 10.51; 95% CI, 2.86 to 26.92), followed by acute myeloid leukemia (SIR 6.62; 95% CI, 4.85 to 8.83), acute non-lymphocytic leukemia (SIR 6.46; 95% CI, 4.8 to 8.52), oropharynx (SIR 6.24; 95% CI, 2.03 to 14.57), and acute lymphocytic leukemia (SIR 5.26; 95% CI, 1.43 to 13.46). However, AER was the highest for the respiratory system (AER 88.64), followed by digestive system (AER 10.85), and myeloid and monocytic leukemia (AER 7.81). The risk of developing SPM in patients with SCLC and LCNEC are summarized in Table 2.

**Table 2:** Total SPM

Site	All cancers					small cell lung cancer					large cell neuroendocrine cancer				
	O	E	O/E	95% CI	Excess risk	O	E	O/E	95% CI	Excess risk	O	E	O/E	95% CI	Excess risk
All sites	1361	887.49	1.53	1.45–1.62	83.11	1195	804.21	1.49	1.4–1.57	75.28	166	83.27	1.99	1.7–2.32	163.46
All solid tumours	1241	790.41	1.57	1.48–1.66	79.08	1083	716.6	1.51	1.42–1.6	70.58	158	73.81	2.14	1.82–2.5	166.34
Oral cavity and pharynx	30	20.08	1.50	1.01–2.13	1.74	28	18.1	1.55	1.03–2.24	1.91	2	1.97	1.02	0.12–3.68	0.07
Floor of Mouth, and Gum and Other Mouth	13	4.08	3.19	1.7–5.45	1.57	13	3.69	3.52	1.88–6.02	1.79	0	0.39	0.00	0.00–9.54	−0.76
Pharynx	8	2.86	2.79	1.21–5.51	0.9	7	2.58	2.71	1.09–5.58	0.85	1	0.28	3.59	0.09–20.02	1.43
Digestive System	227	165.17	1.37	1.2–1.57	10.85	201	149.43	1.35	1.17–1.54	9.93	26	15.75	1.65	1.08–2.42	20.26
Esophagus	17	9.32	1.82	1.06–2.92	1.35	16	8.39	1.91	1.09–3.1	1.47	1	0.94	1.06	0.03–5.93	0.12
Colon and Rectum	113	84.02	1.34	1.11–1.62	5.09	100	76.28	1.31	1.07–1.59	4.57	13	7.74	1.68	0.89–2.87	10.39
Anus, Anal Canal and Anorectum	8	3.19	2.51	1.08–4.94	0.84	5	2.9	1.72	0.56–4.02	0.4	3	0.29	10.44	2.15–30.5	5.36
Pancreas	46	25.96	1.77	1.3–2.36	3.52	41	23.42	1.75	1.26–2.38	3.39	5	2.54	1.97	0.64–4.59	4.85
Respiratory System	650	144.98	4.48	4.15–4.84	88.64	564	131.35	4.29	3.95–4.66	83.34	86	13.63	6.31	5.05–7.79	142.99
Larynx	20	6.92	2.89	1.77–4.46	2.3	19	6.25	3.04	1.83–4.75	2.46	1	0.67	1.48	0.04–8.26	0.64
Lung and Bronchus	627	136.6	4.59	4.24–4.96	86.07	543	123.79	4.39	4.03–4.77	80.75	84	12.82	6.55	5.23–8.12	140.65
Breast	86	122.17	0.70	0.56–0.87	−6.35	74	112.39	0.66	0.52–0.83	−7.39	12	9.78	1.23	0.63–2.14	4.38
Female Breast	86	121.11	0.71	0.57–0.88	−6.16	74	111.44	0.66	0.52–0.83	−7.21	12	9.67	1.24	0.64–2.17	4.61
Female Genital System	20	47.09	0.42	0.26–0.66	−4.76	19	43.35	0.44	0.26–0.68	−4.69	1	3.74	0.27	0.01–1.49	−5.42
Corpus and Uterus, NOS	4	26.93	0.15	0.04–0.38	−4.02	4	24.8	0.16	0.04–0.41	− 4.01	0	2.13	0.00	0–1.73	−4.21
Male Genital System	59	144.85	0.41	0.31–0.53	−15.07	49	130.36	0.38	0.28–0.5	−15.67	10	14.49	0.69	0.33–1.27	−8.87
Prostate	57	143.35	0.40	0.3–0.52	−15.16	47	129.02	0.36	0.27–0.48	− 15.8	10	14.33	0.70	0.33–1.28	−8.56
Urinary System	117	76.03	1.54	1.27–1.84	7.19	104	68.35	1.52	1.24–1.84	6.87	13	7.68	1.69	0.9–2.89	10.51
Urinary Bladder	68	45.07	1.51	1.17–1.91	4.02	59	40.43	1.46	1.11–1.88	3.58	9	4.64	1.94	0.89–3.68	8.61
Kidney and Renal Pelvis	47	28.99	1.62	1.19–2.16	3.16	43	26.15	1.64	1.19–2.21	3.25	4	2.84	1.41	0.38–3.61	2.3
All Lymphatic and Hematopoietic Diseases	95	75.86	1.25	1.01–1.53	3.36	88	68.46	1.29	1.03–1.58	3.76	7	7.41	0.95	0.38–1.95	−0.8
Lymphoma	24	38.88	0.62	0.4–0.92	−2.61	23	35.14	0.65	0.41–0.98	−2.34	1	3.74	0.27	0.01–1.49	−5.41
Myeloma	5	13.46	0.37	0.12–0.87	−1.49	4	12.12	0.33	0.09–0.84	−1.56	1	1.34	0.75	0.02–4.15	−0.68
Leukemia	66	23.52	2.81	2.17–3.57	7.46	61	21.19	2.88	2.2–3.7	7.67	5	2.33	2.15	0.7–5.01	5.28

*O, observed numbers; E, expected numbers

A significantly increased risk was seen for different malignancies among two histology groups. Patients with SCLC were at excess risk of developing digestive system cancers (SIR 1.35; 95% CI, 1.17 to 1.54) and respiratory system cancers (SIR 4.29; 95% CI, 3.95 to 4.66). Similar risk trends were observed, where patients with pulmonary LCNEC had statistically significant excess risk for the development of the digestive system and respiratory system cancers. In the histology-specific analysis, the risk of the oral cavity and pharynx, urinary system and all lymphatic and hematopoietic diseases was not significantly influenced in LCNEC patients and increased in SCLC cases.

The risk of second cancers following lung HGNEC was higher for women than men (SIR = 1.78 [95% CI = 1.65 to 1.91] versus 1.32 [95% CI = 1.22 to 1.43]), and women had the highest SIR values irrespective of any race. SIR values decreased with age, with the uppermost SIR reported for the youngest (age < 50 years) male cohort (SIR 5.21; 95% CI, 2.92 to 8.60). For men and women, SIR values increased with the year of initial primary lung HGNEC diagnosis (Table 3).

**Table 3:** Standardized incidence ratio (SIR) analysis of SMP in patients with a history of an initial primary lung HGNEC by sex, race, age and year of diagnosis, SEER-18

	Observed	Expected	O/E	95% CI	Excess risk
**Total**	1361	887.49	1.53	1.45–1.62	83.11
**Age and Sex**					
Male					
All Men	619	469.98	1.32	1.22–1.43	59.83
< 50	15	2.88	5.21	2.92–8.60	100.27
50–64	187	106.35	1.76	1.52–2.03	83.72
> 65	417	360.75	1.16	1.05–1.27	39.99
Female					
All Women	742	417.5	1.78	1.65–1.91	101.19
< 50	16	5.38	2.98	1.70–4.83	76.02
50–64	207	107.69	1.92	1.67–2.20	80.9
> 65	519	304.43	1.7	1.56–1.86	116.64
**Sex and Race**					
Male					
White	524	404.86	1.29	1.19–1.41	56.6
Black	61	46.61	1.31	1.00–1.68	60.13
Other	34	18.03	1.89	1.31–2.64	111.65
Female					
White	663	377.18	1.76	1.63–1.90	100.63
Black	61	31.78	1.92	1.47–2.47	107.75
Other	18	8.31	2.17	1.28–3.42	104.01
**Sex and Year**					
Male					
2000–2004	57	85.79	0.66	0.50–0.86	−66.4
2005–2010	230	184.25	1.25	1.09–1.42	47.7
2011–2016	332	199.94	1.66	1.49–1.85	120.29
Female					
2000–2004	63	62.86	1	0.77–1.28	0.3
2005–2010	239	157.77	1.51	1.33–1.72	66.49
2011–2016	440	196.87	2.23	2.03–2.45	162.62

### Race and age at diagnosis

All 3 race groups (white, black, and other) were at increased risk of SPM development (white: SIR 1.52, 95% CI, 1.43 to 1.61; black: SIR 1.56, 95% CI, 1.29 to 1.86; and other: SIR 1.97, 95% CI, 1.47 to 2.59). The risks of SPMs in the respiratory system were elevated across all race groups (Table 4). Whites were found to have a significantly elevated risk of SPM of the floor of mouth (SIR 8.11; 95% CI, 3.50 to 15.98), and oropharynx (SIR 5.90; 95% CI, 1.61 to 15.09). In the black racial subgroup, the risk of an SPM was highest in the adrenal gland (SIR 41.01; 95% CI, 1.04 to 228.51), followed by gum and other mouth (SIR 9.10; 95% CI, 1.10 to 32.89), and esophagus (SIR 4.50; 95% CI, 1.23 to 11.52). In the other racial subgroup, the risk of developing an SPM in the digestive system was not significantly altered (SIR 0.66; 95% CI, 0.21 to 1.54), but their risk of SPMs for oropharynx was markedly increased (SIR 69.80; 95% CI, 1.77 to 388.92).

**Table 4:** Risk of SPM after lung HGNEC, stratified by race

	Observed	Expected	O/E	95% CI	Excess risk
**White**					
All Sites	1187	782.04	1.52	1.43–1.61	81.89
All Solid Tumours	1080	695.27	1.55	1.46–1.65	77.8
Oral Cavity and Pharynx	26	17.87	1.45	0.95–2.13	1.64
Floor of Mouth	8	0.99	8.11	3.5–15.98	1.42
Oropharynx	4	0.68	5.9	1.61–15.09	0.67
Digestive System	195	140.5	1.39	1.20–1.60	11.02
Splenic Flexure	5	1.6	3.13	1.02–7.30	0.69
Pancreas	40	22.39	1.79	1.28–2.43	3.56
Respiratory System	568	127.47	4.46	4.10–4.84	89.08
Breast	78	110.42	0.71	0.56–0.88	−6.56
Female Genital System	17	42.44	0.4	0.23–0.64	−5.14
Male Genital System	46	120.8	0.38	0.28–0.51	−15.13
Urinary System	102	68.89	1.48	1.21–1.80	6.7
Urinary Bladder	62	41.77	1.48	1.14–1.90	4.09
Kidney	36	23.49	1.53	1.07–2.12	2.53
All Lymphatic and Hematopoietic Diseases	84	67.83	1.24	0.99–1.53	3.27
Leukemia	59	21.46	2.75	2.09–3.55	7.59
**Black**					
All Sites	122	78.39	1.56	1.29–1.86	85.42
All Solid Tumours	114	70.91	1.61	1.33–1.93	84.42
Oral Cavity and Pharynx	2	1.55	1.29	0.16–4.67	0.89
Gum and Other Mouth	2	0.22	9.1	1.1–32.89	3.49
Digestive System	27	16.98	1.59	1.05–2.31	19.62
Esophagus	4	0.89	4.5	1.23–11.52	6.09
Respiratory System	54	13.17	4.1	3.08–5.35	79.97
Male Genital System	10	18.97	0.53	0.25–0.97	−17.58
Kidney	7	2.74	2.55	1.03–5.26	8.34
Adrenal Gland	1	0.02	41.01	1.04–228.51	1.91
Leukemia	4	1.48	2.7	0.73–6.91	4.93
**Other (American Indian/AK Native, Asian/Pacific Islander)**					
All Sites	52	26.33	1.97	1.47–2.59	108.64
All Solid Tumours	47	23.6	1.99	1.46–2.65	99.04
Oral Cavity and Pharynx	2	0.62	3.24	0.39–11.7	5.85
Pharynx	2	0.19	10.47	1.27–37.81	7.66
Oropharynx	1	0.01	69.8	1.77–388.92	4.17
Digestive System	5	7.56	0.66	0.21–1.54	−10.82
Respiratory System	28	4.23	6.63	4.4–9.58	100.62
Leukemia	3	0.56	5.4	1.11–15.77	10.34

**Fig. 1:**
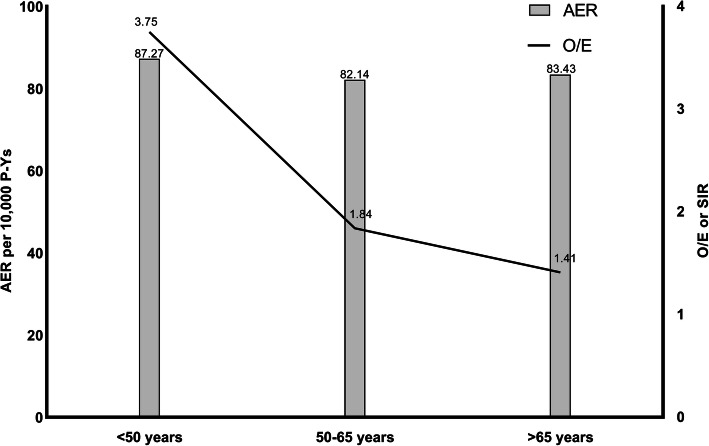
Observed/expected (O/E) incidence and absolute excess rate (AER) for second primary malignancies (SPMs) by patient age at the time of lung HGNEC diagnosis

Overall risk was negatively correlated with age (20–49 years: SIR 3.75, 95% CI, 2.55 to 5.33; 50–64 years: SIR 1.84, 95% CI, 1.66 to 2.03; 65+ years: SIR 1.41, 95% CI, 1.32 to 1.50, Fig. 1). All 3 age groups had an elevated risk of developing SPMs in the digestive system and respiratory system (Table 5). Subgroup analysis suggested that younger patients had an increased risk of SPMs of the pancreas (SIR 41.88; 95% CI, 13.60 to 97.74), floor of mouth (SIR 81.76; 95% CI, 2.07 to 455.51), gum and other mouth (SIR 40.99; 95% CI, 1.04 to 228.4), and respiratory system (SIR 12.41; 95% CI, 4.99 to 25.58). Older patients were at greater risk of malignancies of acute myeloid leukemia (SIR 5.65; 95% CI, 3.87 to 7.98), floor of mouth (SIR 5.48; 95% CI, 1.49 to 14.04), ascending colon (SIR 2.15; 95% CI, 1.35 to 3.25), respiratory system (SIR 3.90; 95% CI, 3.55 to 4.28), and urinary system (SIR 1.37; 95% CI, 1.09 to 1.69).

**Table 5:** Risk of SPM after lung HGNEC, stratified by age

	Observed	Expected	O/E	95% CI	Excess risk
**< 50 years**					
All Sites	31	8.26	3.75	2.55–5.33	87.27
All Solid Tumours	28	7.44	3.76	2.5–5.44	78.9
Oral cavity and pharynx	2	0.25	8.07	0.98–29.16	6.72
Floor of Mouth	1	0.01	81.76	2.07–455.51	3.79
Gum and Other Mouth	1	0.02	40.99	1.04–228.4	3.74
Digestive System	8	1.2	6.66	2.88–13.12	26.09
Colon, Rectum and Anus	3	0.72	4.14	0.85–12.1	8.73
Pancreas	5	0.12	41.88	13.6–97.74	18.73
Respiratory System	7	0.56	12.41	4.99–25.58	24.7
Lung and Bronchus	7	0.49	14.22	5.72–29.3	24.97
Miscellaneous	2	0.09	21.21	2.57–76.62	7.31
**50–64 years**					
All Sites	394	214.04	1.84	1.66–2.03	82.14
All Solid Tumours	361	194.61	1.86	1.67–2.06	75.95
Oral cavity and pharynx	13	6.26	2.08	1.11–3.55	3.08
Floor of Mouth	3	0.37	8.13	1.68–23.76	1.2
Pharynx	4	0.92	4.35	1.18–11.13	1.41
Oropharynx	4	0.27	14.73	4.01–37.71	1.7
Digestive System	62	36.56	1.7	1.3–2.17	11.61
Esophagus	7	2.14	3.27	1.31–6.73	2.22
Small Intestine	4	1.04	3.83	1.04–9.81	1.35
Ascending Colon	6	1.93	3.11	1.14–6.77	1.86
Pancreas	17	4.97	3.42	1.99–5.48	5.49
Respiratory System	186	27.27	6.82	5.88–7.88	72.45
Lung and Bronchus	180	24.94	7.22	6.2–8.35	70.77
Male Genital System	15	36.56	0.41	0.23–0.68	−9.84
Prostate	14	36.09	0.39	0.21–0.65	−10.08
Urinary System	32	14.84	2.16	1.47–3.04	7.83
Urinary Bladder	15	6.92	2.17	1.21–3.57	3.69
Kidney	17	7.4	2.3	1.34–3.68	4.38
All Lymphatic and Hematopoietic Diseases	28	15.75	1.78	1.18–2.57	5.59
Leukemia	24	4.52	5.32	3.41–7.91	8.89
Acute Lymphocytic Leukemia	3	0.24	12.48	2.57–36.48	1.26
Non-Lymphocytic Leukemia	18	2.08	8.67	5.14–13.7	7.27
Acute Non-Lymphocytic Leukemia	16	1.36	11.75	6.72–19.08	6.68
Myeloid and Monocytic Leukemia	18	1.94	9.29	5.51–14.68	7.33
Acute Myeloid Leukemia	14	1.23	11.4	6.23–19.13	5.83
Acute Monocytic Leukemia	2	0.08	24.43	2.96–88.23	0.88
**> 65 years**					
All Sites	936	665.19	1.41	1.32–1.5	83.43
All Solid Tumours	852	588.37	1.45	1.35–1.55	81.22
Oral cavity and pharynx	15	13.55	1.11	0.62–1.83	0.45
Floor of Mouth	4	0.73	5.48	1.49–14.04	1.01
Digestive System	157	127.41	1.23	1.05–1.44	9.12
Colon, Rectum and Anus	97	67.29	1.44	1.17–1.76	9.15
Colon and Rectum	91	65.22	1.4	1.12–1.71	7.94
Colon excluding Rectum	77	49.1	1.57	1.24–1.96	8.6
Ascending Colon	22	10.23	2.15	1.35–3.25	3.62
Respiratory System	457	117.15	3.9	3.55–4.28	104.7
Lung and Bronchus	440	111.17	3.96	3.6–4.35	101.3
Breast	49	82.13	0.6	0.44–0.79	−10.21
Female Genital System	10	30.69	0.33	0.16–0.6	−6.37
Corpus and Uterus, NOS	2	17.05	0.12	0.01–0.42	−4.64
Male Genital System	44	107.8	0.41	0.3–0.55	−19.65
Prostate	43	106.87	0.4	0.29–0.54	−19.68
Urinary System	83	60.72	1.37	1.09–1.69	6.86
All Lymphatic and Hematopoietic Diseases	66	59.42	1.11	0.86–1.41	2.03
Leukemia	42	18.82	2.23	1.61–3.02	7.14
Non-Lymphocytic Leukemia	37	9.31	3.97	2.8–5.48	8.53
Acute Non-Lymphocytic Leukemia	34	6.31	5.39	3.73–7.53	8.53
Myeloid and Monocytic Leukemia	37	8.44	4.39	3.09–6.04	8.8
Acute Myeloid Leukemia	32	5.66	5.65	3.87–7.98	8.11

### Histology

Five hundred and eighty-two patients developed one or more second primary lung cancers (SPLCs). The various histological types of SPLC were assessed within each subset of the lung HGNEC (Table 6). Squamous cell carcinoma was the most common subtype, and a higher proportion was observed following SCLC. Conversely, initial primary LCNECs most presented with SPLC adenocarcinoma (40%). Only 15% of SPLCs were SCLC, which is similar to the incidence of SCLC in the general population. Among the study population, 74% of patients who developed SPLCs initially had regional and distant stage, but only 22% had localized stage. More than half of those SPLCs (55%) presented at advanced or unknown stage, while only 45% had localized disease.

**Table 6:** Distribution of histology and stage in second primary lung cancer (SPLC) patients with history of an initial primary lung HGNEC

	SPLC Histology				Small cell		Other or unknown		Total
	Squamous cell		Adenocarcinoma						
**HGNEC Histology**	N	Row(%)	N	Row(%)	N	Row(%)	N	Row(%)	
SCLC	206	38%	141	26%	81	15%	114	21%	542
LCNEC	20	23%	34	40%	16	19%	15	18%	85
Total	226	36%	175	28%	97	15%	129	21%	627
	**SPLC Stage**								
	Localized		Regional		Distant		Unstaged		Total
**HGNEC Stage**	N	Row(%)	N	Row(%)	N	Row(%)	N	Row(%)	
Localized	71	51%	32	23%	34	24%	3	2%	140
Regional	130	44%	71	24%	86	29%	9	3%	296
Distant	68	41%	40	24%	52	31%	7	4%	167
Unstaged	12	50%	4	17%	7	29%	1	4%	24
Total	281	45%	147	23%	179	29%	20	3%	627

### SPM and latency

**Fig. 2:**
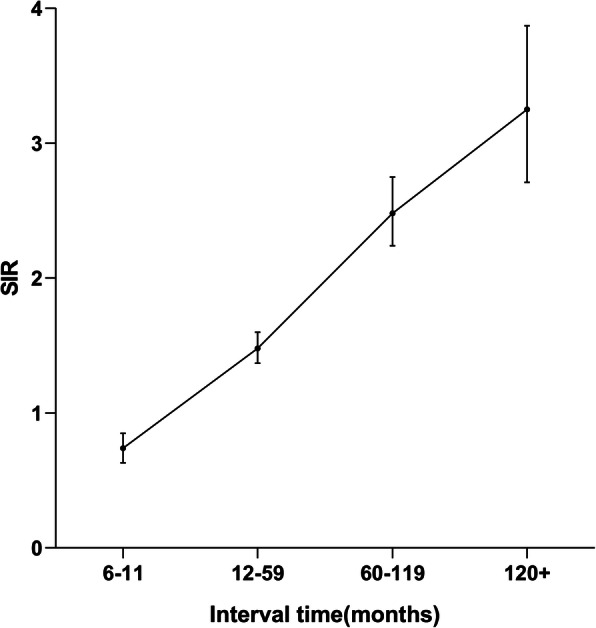
Observed/expected (O/E) incidence (standardized incidence ratio, SIR) of second primary malignancies (SPMs) by latency period after the diagnosis of lung HGNEC over time

The incidence of developing SPMs was relatively high after 12 months of lung HGNEC diagnosis and then increased, with significantly difference from that of the general population (Fig. 2). The risk of oropharyngeal cancer (SIR 9.22; 95% CI, 1.12 to 33.31), and kidney cancer (SIR 2.24; 95% CI, 1.28 to 3.63) was much higher within 6–11 months of the index diagnosis. However, no significant risk of SPM was found in other latency intervals. The risk of mouth floor cancer (SIR 6.90; 95% CI, 1.88 to 17.66), leukemia (SIR 3.84; 95% CI, 2.81 to 5.13), and ascending colon cancer (SIR 2.23; 95% CI, 1.22 to 3.74) was greatly increased within 12–59 months of latency compared to the general population. Significant increases in the risk for cancers of the digestive system and respiratory system also existed 12 months or more after the index diagnosis. The risk of SPM for each latency period is shown in Table 7.

**Table 7:** Risk of SPM after lung HGNEC, stratified by latency

	6–11 months			95% CI	Excess risk	12–59 months			95% CI	Excess risk	60–119 months				Excess risk	120+ months				Excess risk
	O	E	O/E			O	E	O/E			O	E	O/E	95% CI		O	E	O/E	95% CI	
All Sites	174	236.63	0.74	0.63–0.85	−40.5	679	458.53	1.48	1.37–1.6	73.86	380	152.97	2.48	2.24–2.75	241.94	128	39.35	3.25	2.71–3.87	389.58
All Solid Tumours	162	211.36	0.77	0.65–0.89	−31.91	603	408.91	1.47	1.36–1.6	65.02	356	135.52	2.63	2.36–2.91	234.95	120	34.63	3.47	2.87–4.14	375.2
Oral Cavity and Pharynx	9	5.45	1.65	0.75–3.13	2.29	14	10.3	1.36	0.74–2.28	1.24	5	3.42	1.46	0.47–3.41	1.68	2	0.88	2.26	0.27–8.16	4.9
Digestive System	53	43.96	1.21	0.9–1.58	5.84	105	85.1	1.23	1.01–1.49	6.67	51	28.64	1.78	1.33–2.34	23.82	18	7.46	2.41	1.43–3.81	46.33
Respiratory System	34	38.64	0.88	0.61–1.23	−3	317	74.79	4.24	3.78–4.73	81.14	222	25.03	8.87	7.74–10.12	209.9	77	6.52	11.81	9.32–14.76	309.74
Breast	15	30.34	0.49	0.28–0.82	−9.92	39	64.32	0.61	0.43–0.83	−8.48	26	21.86	1.19	0.78–1.74	4.41	6	5.66	1.06	0.39–2.31	1.51
Female Genital System	2	11.64	0.17	0.02–0.62	−6.23	12	24.82	0.48	0.25–0.84	−4.29	5	8.47	0.59	0.19–1.38	−3.7	1	2.17	0.46	0.01–2.57	−5.14
Male Genital System	10	42.93	0.23	0.11–0.43	−21.29	30	74.99	0.4	0.27–0.57	−15.07	12	21.95	0.55	0.28–0.96	−10.6	7	4.97	1.41	0.57–2.9	8.9
Urinary System	28	20.22	1.39	0.92–2	5.03	62	38.71	1.6	1.23–2.05	7.8	23	13.5	1.7	1.08–2.56	10.12	4	3.6	1.11	0.3–2.85	1.76
All Lymphatic and Hematopoietic Diseases	7	19.72	0.35	0.14–0.73	−8.23	61	38.79	1.57	1.2–2.02	7.44	21	13.66	1.54	0.95–2.35	7.82	6	3.7	1.62	0.6–3.53	10.12

### Overall survival and clinical characteristics

Multivariate Cox proportional hazards model was performed to determine risk factors associated with overall survival and cancer-specific survival (Table 8). After adjusting for other factors, patients with regional and distant stage disease were much more likely to have an increased risk of death with HRs of 1.608 (95% CI, 1.317 to 1.964; *P* = 0.000) and 2.113 (95% CI, 1.716 to 2.602; *P* = 0.000), respectively. Patients aged ≥65 years had an elevated risk of death compared with those aged less than 65 years (HR 1.242; 95% CI, 1.085 to 1.422; *P* = 0.002). As for latency time, those patients shorter than 60 months also showed a difference in an elevated risk of death (HR 3.862; 95% CI, 3.310 to 4.507; *P* = 0.000). Beam radiation (HR 1.997; 95% CI, 1.233 to 3.237) was related to the worsening prognosis, but chemotherapy status did not have a significant association with overall survival. Variables that were significantly associated with increased cancer-specific mortality were beam radiation, regional/distant disease, and an interval of < 60 months between the diagnosis of lung HGNEC.

**Table 8:** Cox proportional hazard regression analysis for the overall survival and cancer-specific survival of lung HGNEC patients with SPM

Variable	overall survival			cancer-specific survival		
	HR	95% CI	*P*	HR	95% CI	*P*
Age						
< 65 year		Reference			Reference	
≥ 65 year	1.242	1.085–1.422	0.002	0.981	0.827–1.163	0.824
Sex						
Male		Reference			Reference	
Female	0.962	0.843–1.098	0.569	0.897	0.760–1.059	0.198
Race						
White		Reference			Reference	
Black	0.922	0.727–1.169	0.503	0.870	0.642–1.177	0.366
Other	1.011	0.712–1.434	0.953	1.137	0.747–1.731	0.550
Stage						
Localized		Reference			Reference	
Regional	1.608	1.317–1.964	0.000	1.783	1.373–2.314	0.000
Distant	2.113	1.716–2.602	0.000	2.370	1.809–3.103	0.000
Unknown/unstaged	1.230	0.835–1.811	0.295	1.120	0.661–1.898	0.674
Radiation						
None/Unknown		Reference			Reference	
Beam radiation	1.997	1.233–3.237	0.005	2.254	1.299–3.909	0.004
Other radiation	0.991	0.833–1.178	0.914	0.950	0.763–1.182	0.643
Chemotherapy						
Yes		Reference			Reference	
None/Unknown	0.881	0.715–1.085	0.232	0.915	0.704–1.190	0.509
Latency						
≥ 60 months		Reference			Reference	
6–59 months	3.862	3.310–4.507	0.000	3.761	3.093–4.573	0.000

## Discussion

As far as we know, this study is the first to quantify the occurrence of SPMs after pulmonary HGNEC. Our study revealed that the overall risk of SPM in patients with pulmonary HGNEC was statistically higher than that in the general population. In total, the incidence of SMPs in patients with pulmonary HGNEC is approximately 1.53%. The incidence of SPMs in patients with SCLC is 1.41%, whereas the incidence in patients with lung LCNEC is 3.98%. Going beyond prior researches, we estimated the risk of second malignancies by calculating SIRs, which were stratified by age, sex, race, latency, and histology.

A significantly elevated risk of cancer in pulmonary HGNEC was also evident in our report, especially in patients aged less than 50 years, females, other races (American Indian/AK Native, Asian/Pacific Islander), patients with longer latency periods and LCNEC patients. The SIRs in females were found to be higher than their male counterparts, even though pulmonary HGNEC are less common in the female than male. It is estimated that the incidence of SCLC varied by gender, with a lower frequency in females. Survival was superior to women, indicating genomic incompatibility between the sexes [6]. Carcinogens in cigarette smoke have been hypothesized to preferentially bind to estrogen receptors, thereby inhibiting their carcinogen activation reactions [7]. Furthermore, it has been shown that the use of hormone replacement therapy decreased lung cancer risk in females, especially in female never smokers [8]. Females may be more likely to survive longer and have access to develop an SPM. These factors may potentially explain the higher SIRs and the lower risk of females. However, younger males had the highest SIR. This may be relevant to the declining overall cancer incidence among younger males. A review of the existing studies shows that there are twice as many women as men in younger cancer patients [9]. Thus, the difference between the observed and expected risk of developing cancers in younger males will be greater. Furthermore, the incidence of SPMs increased with age. These results confirmed those of Deng et al. who found increased age as a negative survival predictor in patients with LCNEC [10].

We observed that lung HGNEC survivors, particularly SCLC survivors, were less likely to develop cancers of the breast, female genital system, and prostate. In contrast, patients with lung HGNEC had elevated risks of getting leukemia and cancers of the oral cavity and pharynx, colon and rectum, esophagus, pancreas, urinary bladder, kidney and renal pelvis, and lung and bronchus. Cancer risk reduction in these patients is consistent with prior researches, which are relevant to lung cancer and non-small cell lung cancer [11, 12]. Although the causes of risk reduction are not well understood, they may be associated with patient age at diagnosis of SCLC. The current incidence of SCLC was highest in the 65–79 age group, and the number of SCLC patients decreased in most age groups over the past few decades, primarily because of public awareness about smoking and comprehensive tobacco control programs [6]. Nevertheless, older patients who have SCLC may not have an equal opportunity for an SPM as the total population of the United States. Conversely, the increased rate of certain cancers following primary lung HGNEC seems to be attributed to smoking. Lung HGNEC patients had a greater risk of developing respiratory system cancer in all age groups. This correlation was also evident in a subgroup analysis of the younger populations below the age of 50 years.

Other considerations are more deliberate surveillance and molecular mutation. Once patients are newly diagnosed with cancer, they may receive more monitoring. In most lung HGNEC, only a few genes were found to be mutated regularly. Tumour suppressor protein 53 (TP53) and retinoblastoma 1 (RB1), which are strongly associated with smoking, are mutated in nearly all SCLC [13, 14] and most of these lung LCNEC [15]. Even so, no targeted therapy could be translated from basic research to standard treatment until now. Smoking is also a risk factor for HPV-negative head and neck squamous cell carcinoma. Mutations are more frequent in these tumours from smokers than non-smokers. TP53 mutations are more common in HPV-negative tumours and have been related to poor survival and therapeutic resistance [16]. This may be relevant to the increased rate of the oral cavity and pharynx cancer observed in our SCLC cohort, so patients with SCLC would necessarily be expected to have closer surveillance for these smoking-related malignancies. Many studies showed that acute myeloid leukemia and lung HGNEC have the same c-Kit high expression. Positivity expression of c-Kit is observed in 49% of LCNEC and 47% of SCLC cases [17], and the frequency of positive c-Kit among acute myeloid leukemia was about 80% [18]. However, there is no evidence to suggest that these two tumours are closely linked. Similarly, the relationship between lung HGNEC and acute myeloid leukemia has not been covered.

In lung cancers following pulmonary HGNEC, 74% of SPLCs were found to be adenocarcinoma and squamous cell carcinoma. Much higher rates of squamous cell carcinoma were detected relative to the SCLC subset. To our knowledge, squamous and small cell histology are the most strongly related to smoking. This study supports our understanding of SCLC most closely linked to smoking [19]. Interestingly, lung cancers following lung LCNEC were much more likely to be of adenocarcinoma histology. However, based on currently observed data, we cannot identify the relationship between these two cancers. Thus, there is a need for advanced assessment techniques such as gene expression to provide information for patients and clinicians.

Multivariate Cox regression model revealed that older patients, advanced historical stage, beam radiation history, and shorter latency time were associated with increased risk of developing the SPMs in lung HGNEC patients. Our data found a higher risk of developing the SPMs to be in those aged more than 65. As expected, this is likely because older patients have a higher probability of developing invasive cancer. In addition, lung HGNEC has a high risk of death in the regional and distant stage. Beam radiation was strongly associated with increased overall mortality. A study demonstrated that radiotherapy, in combination with chemotherapy has been described as having an additive effect on the occurrence of secondary cancer [20]. However, our paper could not confirm this finding. Currently, there is no report about the long-term cause of death in patients with lung HGNEC. We also use the HR of cancer-specific survival to determine the impact of SPM on pulmonary HGNEC patients. The risk of cancer-specific mortality did not increase with age. This is because older patients are more likely to die of other diseases such as cardiovascular disease.

The advantages of this research include a large sample size, which strengthens the generalizability of the findings. However, there are some limitations of the current study. The cross-sectional limitations rely on the retrospective nature and inherent limitations of publicly-accessible databases, such as the lack of treatment details (radiation dosage, surgery type), family history, and lifestyle factors (smoking). Half of the data in some variables, such as grade, is unknown. These factors would affect the comprehensive analysis of risk factors for SPM development. Also, our analysis may have missed patients who change their living place throughout the follow-up period. Once they have SPMs, these details were not registered in the SEER database. Finally, germline mutations are not provided by the SEER database. Further research is required to identify the appropriate screening/surveillance recommendations and to clarify potential genetic factors that may lead to increased cancer risk for these patients.

## Conclusions

The risk of SPM is significantly higher among lung HGNEC than the U.S. general population. The most common and biologically meaningful were acute monocytic leukemia, acute myeloid leukemia, and floor of mouth tumours, but an elevated risk for lung and oropharynx cancers was also demonstrated. Old age, advanced stage, beam radiation, and shorter latency time were identified as negative prognostic factors. Chemotherapy did not substantially influence the incidence of SPMs, which can be traced to the lack of adequate data. The observed increased risk may be explained by genetic susceptibility and lifestyle modifications. With the ongoing improvement in the long-term survival of patients with lung HGNEC, evaluation for SPMs will become even more crucial in the follow-up care of these patients.

## Data Availability

The datasets used and/or analyzed during the current study are available from the Surveillance, Epidemiology, and End Results (SEER) database (http://seer.cancer.gov/data/sample-dua.html).

## Abbreviations

HGNEC: High-grade neuroendocrine carcinoma
SPM: Second primary malignancy
SEER: Surveillance, Epidemiology and End Results
SIR: Standardized incidence ratio
AER: Absolute excess risk
SLCL: Small cell lung cancer
LCNEC: Large cell neuroendocrine carcinoma
OS: Overall survival
CSS: Cancer-specific survival
HR: Hazards ratio
CI: Confidence interval
SPLC: Second primary lung cancer
TP53: Tumour suppressor protein 53
RB1: Retinoblastoma 1
